# Oxygen as a Master Regulator of Human Pluripotent Stem Cell Function and Metabolism

**DOI:** 10.3390/jpm11090905

**Published:** 2021-09-10

**Authors:** Kinga Nit, Malgorzata Tyszka-Czochara, Sylwia Bobis-Wozowicz

**Affiliations:** 1 Department of Cell Biology, Faculty of Biochemistry, Biophysics and Biotechnology, Jagiellonian University, 30-387 Krakow, Poland; kinga.polanska@doctoral.uj.edu.pl; 2 Faculty of Pharmacy, Jagiellonian University Medical College, 30-688 Krakow, Poland; malgorzata.tyszka-czochara@uj.edu.pl

**Keywords:** oxygen, hypoxia, hypoxia-inducible factors, induced pluripotent stem cells, metabolism, cancer stem cells

## Abstract

Human-induced pluripotent stem cells (hiPSCs) offer numerous possibilities in science and medicine, particularly when combined with precise genome editing methods. hiPSCs are artificially generated equivalents of human embryonic stem cells (hESCs), which possess an unlimited ability to self-renew and the potential to differentiate into any cell type of the human body. Importantly, generating patient-specific hiPSCs enables personalized drug testing or autologous cell therapy upon differentiation into a desired cell line. However, to ensure the highest standard of hiPSC-based biomedical products, their safety and reliability need to be proved. One of the key factors influencing human pluripotent stem cell (hPSC) characteristics and function is oxygen concentration in their microenvironment. In recent years, emerging data have pointed toward the beneficial effect of low oxygen pressure (hypoxia) on both hiPSCs and hESCs. In this review, we examine the state-of-the-art research on the oxygen impact on hiPSC functions and activity with an emphasis on their niche, metabolic state, reprogramming efficiency, and differentiation potential. We also discuss the similarities and differences between PSCs and cancer stem cells (CSCs) with respect to the role of oxygen in both cell types.

## 1. Introduction

Stem cells (SCs) are a peculiar type of cells that possess the ability to self-renew and differentiate into other cell types. Based on their differentiation potential, five basic groups of SCs can be distinguished: (1) totipotent SCs with the ability to differentiate into every cell type, including the placenta; (2) pluripotent stem cells (PSCs), which can differentiate into cells from all three germ layers (ectoderm, mesoderm, endoderm), excluding the placenta; (3) multipotent SCs, which give rise to the cells from only one germ layer, e.g., hematopoietic SCs, mesenchymal SCs, neural SCs; (4) oligopotent SCs, also called progenitors, which can differentiate into several types of cells of the same origin; (5) unipotent SCs, capable of forming one specific cell type. Totipotent and pluripotent SCs are present only in the early stages of embryonic development, whereas multipotent, oligopotent, and unipotent SCs reside in adult tissues [1]. Owing to their properties, SCs attract a lot of attention as a cell source for both research and clinical purposes. Among them, PSCs constitute particularly useful tools for designing tailor-made medical therapies, since they can replenish any tissue or organ with a desired cell type.

PSCs that naturally exist in the early stages of embryonic development are called embryonic stem cells (ESCs). After fertilization, the zygote divides to give rise to morulae, which are composed of totipotent stem cells. The cells continue to divide and begin to differentiate, forming the blastocyst. PSCs of the inner cell mass (ICM) or the epiblast of the blastocyst constitute ESCs [2]. Despite their extensive differentiation potential, human ESC (hESC) use in research and medicine raises many ethical issues due to their origin and the necessity to destroy an embryo. Therefore, the generation of induced PSCs (iPSCs) in 2006 from mouse somatic cells and a year later from human cells by Shinya Yamanaka’s group solved the ethical concerns associated with the use of hESCs and opened up numerous possibilities in science and medicine [3,4].

iPSCs were first obtained by reprogramming somatic cells with transcription factors that are crucial to the maintenance of pluripotency: OCT4 (octamer-binding transcription factor 4, also known as POU domain class 5 transcription factor 1; POU5F1), SOX2 (SRY (sex determining region Y)-box 2), KLF4 (Kruppel-like factor 4), c-MYC (cellular myeolocytomatosis oncogene), which are collectively called Yamanaka factors [3,4]. Since then, the number of protocols and the variety of reprogramming factors used to generate iPSCs have expanded [5], making them a novel and exciting alternative to ESCs. Firstly, iPSCs may serve as a model to study embryonic development and cell differentiation mechanisms [6,7]. Secondly, iPSCs derived from a human patient affected by a specific disease enable drug testing and disease modeling [8,9,10]. Thirdly, human iPSCs (hiPSCs) may serve as an excellent cell source in a personalized therapy [11]. Importantly, the application of genome editing tools such as the CRISPR/Cas9 (clustered regularly-interspaced short palindromic repeats/CRISPR-associated protein 9) system [12,13] to hiPSCs allows the determination of the functions of specific genes and discovery of the mechanisms underlying human diseases. Going a step further, the use of gene-corrected hiPSCs derived from a patient suffering from a genetic disease may accelerate the development of new therapies [14].

Despite the abovementioned advantages of using hiPSCs in biomedical studies, there are still some hurdles to be overcome to ensure their safety and reliability. One of these concerns relates to the process of reprogramming, which may induce genetic mutations [15]. Other obstacles include epigenetic memory in iPSCs [16], teratoma formation in vivo [17], and genetic instability, particularly in the long-term in vitro culture [18]. Importantly, the functions and activity of hiPSCs might be strongly influenced by the cellular microenvironment. Accordingly, oxygen tension seems to be one of the key factors modulating cellular metabolism and pluripotency [19,20,21,22].

In this review, we present recent data on the role of oxygen in various aspects of hPSC functioning, with emphasis on cellular metabolism. First, we present the basic features of PSCs. Next, we discuss the role of hypoxia in the PSC niche. Furthermore, we focus on the influence of oxygen on PSC metabolism and regulation of reprogramming and differentiation. Finally, we address the role of hypoxia in cancer stem cells (CSCs), which share many common features with PSCs.

## 2. Basic Features of PSCs

**Figure 1:**
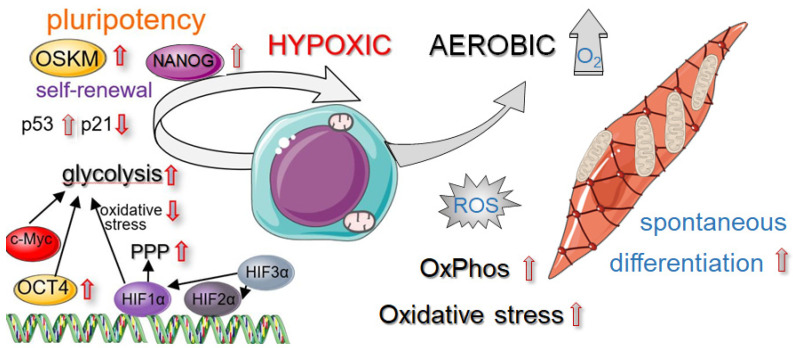
Basic aspects of pluripotent stem cell (PSC) biology. PSCs, which reside in hypoxic niches (3–5% O_2_), maintain pluripotency and self-renewal capacity. Atmospheric oxygen level (21% O_2_) may increase mitochondrial OxPhos with higher ROS production and increased spontaneous differentiation. Abbreviations: OKSM—Yamanaka’s factors: OCT4, KLF4, SOX2, c-MYC; HIF—hypoxia-inducible factor; OxPhos—oxidative phosphorylation; PPP—pentose phosphate pathway; ROS—reactive oxygen species.

The exceptional ability to self-renew, which is a hallmark of PSCs, is governed by the expression of three core transcription factors, OCT4, SOX2, and NANOG (Figure 1). Among others, OCT4 is considered as the major factor responsible for maintaining cell pluripotency. Additionally, it represses the expression of genes that are involved in cell differentiation [23]. In contrast, c-MYC and KLF4 may exert an opposite effect on cells. It was shown that overexpression of c-MYC vividly increases the proliferation rate via induction of p53 activity, while KLF4 restrains the cell cycle by activation of the p21 regulator. Therefore, the precisely established equilibrium between transcription factors in PSCs controls their fate. Moreover, the level of KLF4 expression is inversely correlated with the extent of DNA damage and p53-mediated apoptosis [24].

The expression pattern of transcription factors is reflected in the cellular metabolism in naïve and primed PSCs. Within the cell, mitochondria are factories for energy production via oxidative phosphorylation (OxPhos). In naïve PSCs, which correspond to a more primitive state of embryogenesis and are characterized by X chromosome reactivation in female cells [25], the number of mitochondria is relatively low. At this stage, the cells are bivalent and dynamically switch between OxPhos and glycolysis for energy production [26]. When PSCs are activated and start to proliferate by self-renewing (and without differentiation), the mitochondrial capability is not enough to meet the bioenergetic demands of accelerated growth. Therefore, in primed PSCs, the number of mitochondria does not increase, and the cells generate ATP molecules via glycolysis. It was reported that activation of OxPhos is implemented at the initial stages of differentiation of PSCs [27,28]. Growing evidence indicates that oxygen abundance and metabolic pathways in PSCs are tightly interconnected (Figure 1).

## 3. Hypoxic Niche of PSCs

### 3.1. Oxygen Concentration Impacts the PSC Niche

One of the crucial factors determining SC function is an appropriate environment, which is called an SC niche. It is defined as the physiological microenvironment in a specific location in the organism occupied by SCs. It supports and enables basic functions of SCs, such as self-renewal and differentiation. The SC niche consists of several key components encompassing: (i) the extracellular matrix with a basement membrane and long biopolymers, e.g., vitronectin, laminin; (ii) other cells, e.g., neighboring cells in the tissue, immune cells, neurons, cells forming blood vessels; (iv) soluble signaling factors, including growth factors, hormones, cytokines, chemokines; (v) biophysical factors, such as elasticity and stiffness; and (vi) environmental factors, including metabolites, temperature, and oxygen level [29]. While each of the niche components can strongly influence SC physiology, a growing body of evidence supports the crucial effect of oxygen concentration on various types of SCs, including mesenchymal SCs, hematopoietic SCs, and neural SCs [30,31,32,33,34]. Similarly, oxygen has been indicated as a master regulator of PSC functions, influencing cellular reprogramming, differentiation, genetic stability, and the maintenance of pluripotency [20,35,36,37,38].

### 3.2. Low Oxygen Condition during Embryonic Development

The induction of pluripotency in somatic cells occurs mainly in in vitro cultures; therefore, iPSCs do not possess an in vivo biological niche. However, they are considered functional equivalents of hESCs, which naturally exist during human embryogenesis. Therefore, the current knowledge about the PSC niche is based on the niche of hESCs. Although ESCs are present only transiently during development as they differentiate into other cell types after implantation of the blastocyst, their niche has already been characterized [39]. Some of the most important components of the ESC niche are neighboring cells, the presence of which are essential for epiblast development and proper differentiation. The interplay between ESCs and neighboring cells via cell position and cell signaling is a major factor controlling gene expression in each cell type and guiding proper embryo development. However, oxygen tension may significantly impact ESC function and differentiation [19,36,40].

For ethical reasons, it is not possible to determine the exact oxygen concentration at which the human blastocyst develops. However, numerous animal studies have been conducted to address this question [41,42,43,44]. Measurements in oviducts and uteri of rabbits, hamsters, and rhesus monkeys show that oxygen tension during early embryonic development ranges from 11 to 37 mm Hg (1.5–5.3% O_2_) [32]. Such a reduced oxygen level is termed hypoxia, as opposed to an ambient oxygen condition (21% O_2_), which is called normoxia. Yet it is important to emphasize that such an environment with low oxygen abundance constitutes the physiological condition for PSCs. Moreover, studies on mouse embryos have shown that a culture of zygotes in 5% oxygen improved ICM development in the blastocyst, compared to 20% O_2_ [43]. Consistent with these results, oxygen tension affected the number of ICM cells in bovine blastocysts, with the highest number obtained when cultured at 2% O_2_, followed by 7 and 20% of oxygen [45].

### 3.3. Hypoxia Affects hPSCs Culture In Vitro

**Figure 2:**
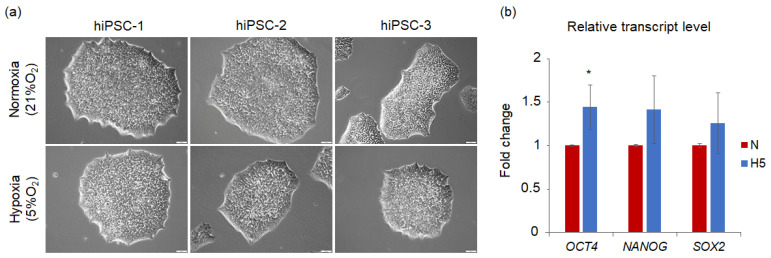
Morphology and expression level of pluripotency-associated genes in three hiPSC lines, cultured in either normoxia (21% O_2_; N) or hypoxia (5% O_2_; H5). hiPSCs were continuously cultured in respective oxygen conditions for 4 passages, in StemFlex (Gibco), on Geltrex (Gibco)-coated dishes. (**a**) Representative pictures of hiPSC colonies were acquired with the Leica DMI6000B v. AF7000 microscope (Leica Microsystems). Scale bar is 50 µm; (**b**) relative expression levels of *OCT4*, *NANOG*, and *SOX2* genes in hiPSCs measured with the ΔΔCt method using *18SRNA* as a calibrator. The graph shows the mean ± SD. Student’s *t*-test was used to compare the expression between the groups. * Statistical significance at *p* < 0.05 is indicated with an asterisk.

Accumulating evidence suggests a significant role of oxygen concentration in in vitro cultures of PSCs. It has been demonstrated that physiological hypoxia (3–5% O_2_) supports an undifferentiated state of hPSCs, and decreases the rate of spontaneous differentiation, which can be observed as areas of flattened and enlarged cells with translucent cytoplasm localized at the periphery of the colonies. Such areas are smaller or absent under hypoxic conditions, compared to normoxia (21% O_2_) [19,20,36,40,46]. Importantly, hypoxia was crucial to the maintenance of inactivated X chromosomes in hESCs, which is an indicator of naïve pluripotency [47]. However, the effect of low oxygen tension on such parameters as cell growth and the expression of pluripotency markers differs among various studies, as summarized in Table 1. Most of the available data show accelerated cell growth under hypoxic conditions [19,20,37,40] and enhanced expression of pluripotency markers, *OCT4*, *SOX2*, *NANOG* [20,40,48]. Our own results stayed in line with these observations, supporting the beneficial role of a low oxygen condition in the maintenance of pluripotency in hiPSCs (Figure 2). However, no impact or even a decrease in the proliferation rate or gene expression level of the core transcription factors was observed [36,46,49,50]. Such discrepancies may occur for several reasons, including the duration of hypoxic treatment, cell medium composition, presence of a feeder layer, and method of cell culture (hypoxic chamber vs. hypoxic incubator), which all influence cellular metabolism and affect cell fate.

## 4. Influence of Oxygen on iPSCs Metabolism

In conditions of oxygen abundance in the cellular milieu, the somatic cells generate energy mostly using mitochondrial respiration via the OxPhos process. OxPhos refers to a series of redox reactions in the mitochondrial matrix, resulting in a highly efficient production of large amounts of adenosine triphosphate (ATP; 24–28 ATP molecules are generated per one glucose molecule converted into pyruvate). When the oxygen level drops, the energetic demand of the cell may be satisfied by rapidly operating glycolytic reactions. In contrast to OxPhos, glycolysis is a less efficient source of energy (only two net ATP molecules per one glucose molecule are produced via substrate-level phosphorylation), and it can generate both anabolic intermediates for cellular growth and ATP. Importantly, energy is provided very rapidly to satisfy the cell’s needs, owing to the much higher speed of glycolysis reactions [51,52]. Considering the significant role of the low oxygen condition in regulating PSC function, it is important to understand how these processes operate in PSCs and whether resulting metabolic changes may be useful to manipulate PSC biology.

### 4.1. Glycolysis Promotes Pluripotency and Self-Renewal in PSCs

**Figure 3:**
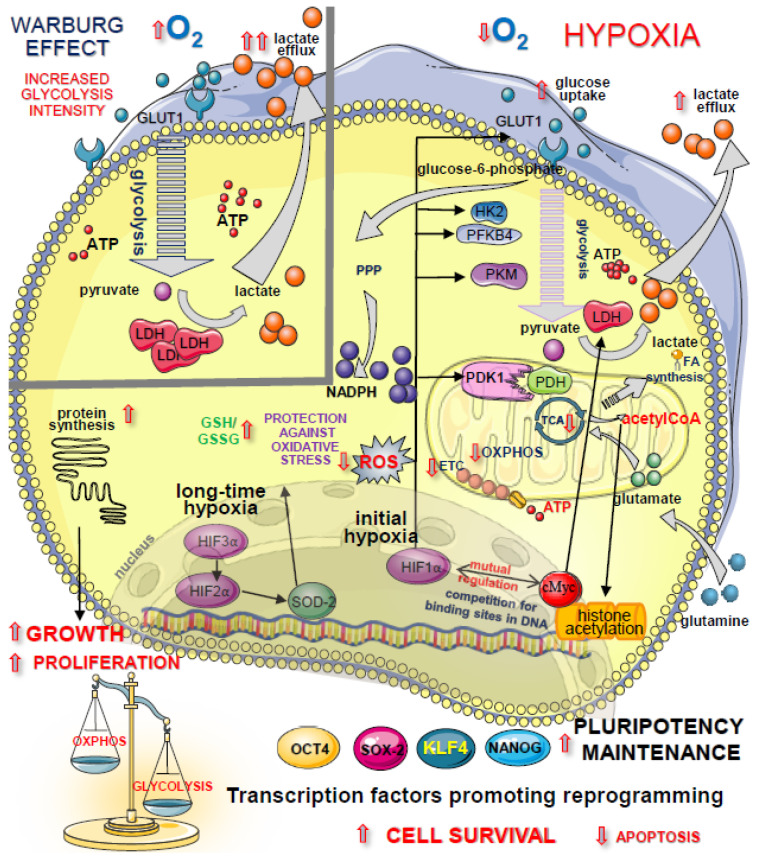
Outline of the cellular metabolism under hypoxic conditions. Both transcription factors, HIF1α and HIF2α, inhibit mitochondrial glucose consumption by restraining pyruvate transport to mitochondria, with less ROS as a by-product and abundant ATP synthesis from glycolytic pathways. HIF1α governs the expression of transcripts for glucose transporter GLUT1 and glycolytic enzymes (HK2, PFK). High *c-MYC* expression increases the catabolism of glucose to lactate and glutamine flux into the TCA cycle as well as increases protection against oxidative stress via PPP (by production of reduced NADPH for glutathione regeneration). HIF3α facilitates HIF2α transcription and curbs HIF1α expression. Upper part: the comparison of the intensity of glycolytic flux under hypoxia (low O_2_ concentration; right panel) and during “Warburg effect” (O_2_ concentration in the air; left panel). Abbreviations: ATP—adenosine triphosphate; ETC—electron transport chain; FA—fatty acids; GLUT1—glucose transporter 1; GSH/GSSG—the ratio of reduced glutathione (GSH) to oxidized glutathione (GSSG); HIF—hypoxia-inducible factor; HK2—hexokinase 2; LDH—lactate dehydrogenase; NADPH—nicotinamide adenine dinucleotide phosphate; OXPHOS—oxidative phosphorylation; PDK1—pyruvate dehydrogenase kinase 1; PFKB4—6-phosphofructo-2-kinase/fructose-2,6-biphosphatase 4; PKM—pyruvate kinase muscle isoenzyme; PPP—pentose phosphate pathway; ROS—reactive oxygen species; SOD2—superoxide dysmutase 2; TCA—tricarboxylic acid.

As mentioned, PSCs rely mostly on glycolysis as an energy producing pathway, in contrast to somatic cells, which utilize OxPhos for ATP production. The recent study of Lees et al. using the uptake of ^13^C-labeled glucose demonstrated that an oxygen level fixed at 5% increases the intracellular glycolytic flux in hPSCs [53]. In contrast, 20% oxygen in the environment promotes intermediate production in the tricarboxylic acid (TCA) cycle and the activity of mitochondria for ATP generation [53]. It was found that PSCs prefer high-glucose catabolism. In particular, ESCs generate energy mostly by glycolysis, even in oxygen abundance. This phenomenon is called the “Warburg effect” and is also typical of cancer cells. Moreover, the activation of the extensive transcriptional program under hypoxia occurs, and this feature is shared by many types of SCs, even if some differences exist between particular lineages [30]. A common strategy of highly proliferating cells—a switch from mitochondrial OxPhos to glycolytic metabolism—aims at meeting the elevated bioenergetics requirements (Figure 3). In compliance, during the intensive growth, the activation of glycolysis supports the rapid generation of ATP and provides cells with macromolecular nucleotide precursors and intermediates for the synthesis of nonessential amino acids. In fact, both metabolic advantages are necessary for sustaining an increased proliferation rate in PSCs [51].

The glycolytic flux is primarily limited by glucose uptake into the cells via GLUT receptors (solute carrier family 2 member receptors, SLC2A). Then, a rapid phosphorylation of glucose to glucose 6-phosphate occurs to arrest the glucose molecule within the cell. The latter step is rate-limiting and depends on the ATP pool, which links cellular energetics to the efficacy of glucose transportation. The excessive glucose carbons not utilized for biosynthesis are preferentially converted to lactate. This preserves a pool of an oxidized form of nicotinamide adenine dinucleotide (NAD^+^) at a sufficient level to sustain glycolysis and avoid supplying the mitochondria with a reduced NAD form—NADH. Since NADH is a by-product of the TCA cycle, it may curb the activity of mitochondrial processes. In such a way, an augmented glucose uptake and catabolism under hypoxia result in enhanced production of lactate, which is subsequently removed from the cell as a redundant by-product. Up to 70% of glucose is converted to lactate in PSCs [53]. The metabolic pathways are reprogrammed in iPSCs, compared to somatic cells, leading to significantly lower consumption of oxygen, a typical feature of PSCs [54]. In contrast, inhibition of glycolysis impedes pluripotency in these cells [28].

The enhanced glucose catabolism may operate in highly proliferating cells, even in the abundant presence of oxygen in the cellular environment. This metabolic profile was identified as typical for cancer cells, and, as already mentioned, was recognized as a core feature of the Warburg effect [55]. The metabolic profile of such cells was extensively described by Hanahan and Weinberg [56] and Pavlova et al. [57]. Like anaerobic glycolysis, the Warburg effect enables the rapid formation of biomolecules and ATP for cell proliferation (Figure 3, left upper panel). Moreover, Tohyama et al. showed that in proliferating, hiPSC intermediate formation in the TCA cycle from pyruvate carbons may be altered due to the impaired expression of two enzymes, aconitase 2 (ACO2) and isocitrate dehydrogenase 2/3 (IDH2/3) [58]. Under such conditions, glutamine carbons (entering the TCA cycle as α-ketoglutarate, α-KG) become important players to sustain the TCA cycle and satisfy the anabolic demands of highly proliferating PSCs [59].

### 4.2. HIFs Mediate the Transcriptional Response to Low Oxygen Tension via Upregulation of Glycolysis

During hypoxic conditions, the cells activate numerous adaptive responses to coordinate the modest O_2_ supply with the metabolic, bioenergetic, and redox requirements of proliferating cells. The major sensors of low oxygen conditions in cells are hypoxia-inducible factors (HIFs). HIFs are transcription factors that act as heterodimers consisting of two subunits: α and β. Upon dimerization, HIFs can translocate to the nucleus, bind to specific sequences called hypoxia-response elements (HREs), and activate transcription of hypoxia-regulated genes. While the HIF-β subunit is present in cells independent of oxygen concentration, the stability of the HIF-α subunit is regulated by the intracellular oxygen level [60]. Under normal oxygen conditions, the HIF-α subunit is hydroxylated by the prolyl hydroxylase domain-containing enzymes (PHDs), which then allows ubiquitination by the von Hippel–Lindau (vHL) tumor suppressor protein. Marking by ubiquitin directs the HIF-α subunit to proteasomes for degradation. However, under hypoxic conditions (1–5% O_2_), PHD activity, which relies on oxygen as a substrate for enzymatic reaction, is substantially reduced. As a consequence, the HIF-α subunit is stabilized and undergoes heterodimerization with the β-subunit [61]. So far, three types of HIF-α subunits have been described—HIF1α, HIF2α, and HIF3α—which differ in both oxygen reactivity and exerted functions [62].

HIF1α is commonly expressed in almost all cell types and acts as a master regulator of numerous hypoxia-inducible genes. Importantly, the functional interaction of HIF1α with other transcription factors results in the final effect on the expression of target genes to promote cell survival. Oxygen deprivation in HIF1α-deficient cells leads to energy stress, an elevated level of reactive oxygen species (ROS), and subsequent cell death due to apoptosis. On the other hand, mitochondria may act as cellular oxygen sensors and with the induction of ROS they affect PHDs, which, as previously mentioned, facilitate HIF1α proteasomal degradation [63].

The concerted action of hypoxic conditions/HIF1α and the oncogene c-MYC regulates various aspects of cellular metabolism essential for the maintenance of a high proliferation rate and prevention of apoptosis in PSCs. This includes the upregulating of glucose transporter 1 (GLUT1) and several glycolytic enzymes, such as hexokinase (HK2), and phosphofructokinase (PFK) isoenzymes, such as 6-phosphofructo-2-kinase/fructose-2,6-biphosphatase 4 (PFKB4). The high expression of c-MYC in the inner cell mass of a blastocyst in vivo promotes self-renewal and pluripotency of hESCs [51]. Accordingly, the study of Varlakhanova et al. using mESCs revealed that *c-Myc* knockout alleviates both processes, glycolytic catabolism, and self-renewal [64]. On the contrary, the ectopic expression of *c-MYC* significantly enhances the glycolysis pathway. Under hypoxic conditions, the catabolism of glucose to lactate increases even more because of the strong upregulation of lactate dehydrogenase (LDH) [59]. Additionally, HIF1α directs glucose to a nonoxidative pentose phosphate pathway (PPP), which promotes the generation of intermediates for nucleotide synthesis and funnels glucose carbon away from mitochondria. Consequently, oxygen consumption by the cells is suppressed. The repression of pyruvate flux to acetyl-CoA occurs via upregulation of the HIF1α target gene, pyruvate dehydrogenase kinase 1 (PDK1). Thereby, overexpression of PDK1 reduces ROS formation and promotes cell survival under hypoxia. Moreover, increased PDK1 activity supports glycolysis, a major contributor to ATP synthesis (Figure 3).

Importantly, the HIF1α-dependent metabolic shift promotes cell viability during the first stage of hypoxic stress. After 48 h of oxygen deprivation, the regulation of the cellular metabolism is dominated by another member of the HIF family, HIF2α. Data from various studies indicate HIF2α as a critical factor governing the expression of pluripotency-associated genes in hPSCs in response to hypoxia [19,20,40]. Similar to HIF1α, HIF2α exerts its effect by the inhibition of glucose-derived carbon consumption in mitochondria. This occurs via the induction of the expression of an isoenzyme PDK4, and also results in the inhibition of oxidative stress. Such a metabolic state is achieved through another mechanism that increases antioxidant capacity, namely, via activation of the expression of the mitochondrial enzyme superoxide dismutase 2 (SOD2) [63]. While HIF1α and c-MYC may compete for binding sites of target genes, the elevated HIF2α level promotes c-MYC activity in a variety of cell lines and in cancer stem cells (CSCs) [30,65]. Under prolonged oxygen deprivation, *HIF3α* also becomes transcriptionally active. Forristal et al., demonstrated that HIF1α and HIF3α may negatively regulate each other’s expression in hESCs [40]. Importantly, HIF3α regulates the long-term response to hypoxia by increasing the endogenous expression of transcription factors such as OCT4. It was shown that the knockdown of HIF2α or HIF3α, but not HIF1α, leads to a drop in the expression of OCT4, NANOG, and SOX2 in hypoxic conditions (5% O_2_) [30]. Kim et al. showed that these core pluripotency factors regulate the transcriptional activity of genes encoding glycolytic enzymes in mESCs. In particular, it was demonstrated that Oct4 triggers the expression of *Hk2* and the pyruvate kinase muscle isoenzyme (*Pkm)* [52]. In this way, the supportive effect of glycolysis on the proliferation of iPSCs persists. Accordingly, HIF2α depletion was shown not only to decrease the protein expressions of OCT4, SOX2, and NANOG but also to hamper proliferation of hESCs [40]. Thus, HIF2α seems to play a protective role during mammalian embryogenesis. When mouse embryos are kept at the atmospheric O_2_ level, a subsequent increase in oxidative stress and induction of apoptosis occurs. In contrast, the number of apoptotic cells in blastocysts decreases when embryos are cultured in 3% O_2_ [66]. Prolonged hypoxia may promote an HIF-dependent state of resistance to apoptosis since restrained mitochondrial biogenesis may limit mitochondrial involvement in apoptotic and non-apoptotic cell death [67]. In contrast to PSCs, somatic mammalian cells respond to hypoxic stress by restraining cell growth and inducing cell cycle arrest [65].

## 5. Oxygen on the Road to Pluripotency

### 5.1. Influence of Hypoxia on Reprogramming Efficiency

The available data suggest a positive effect of hypoxia on reprogramming efficiency [27,54]. It was shown that hypoxia (5% O_2_) initiated 6 days after the induction of human fibroblast reprogramming increased the number of PSC colonies up to 4.2-fold, compared to normoxia [35]. However, enhancement of iPSC generation by a hypoxic environment may differ among various cell types. When human dental pulp cells were reprogrammed, hypoxia (3% O_2_) was also beneficial, but at a different stage [22]. In this case, applying a low oxygen condition for 6 days, started at 24 h after retroviral transduction with four transcription factors (OCT3/4, SOX2, KLF4, c-MYC) increased hiPSC colony formation up to 5.1-fold compared to normoxia. In contrast, prolonged exposure to hypoxia (up to 25 days) decreased reprogramming efficiency and impaired colony morphology [22]. Overall, these data suggest that hypoxia improves reprogramming efficiency, although the results may depend on the cell type, the duration of hypoxia, and the stage of reprogramming.

### 5.2. Metabolic Switch during Reprogramming

One of the most important processes that occurs in cells during somatic cell reprogramming is the metabolic switch from OxPhos to glycolysis (Figure 3) [8]. Recent data indicate that such a transition is not a passive process of altering the participation of both pathways in ATP production, but requires active changes at different metabolic states [67,68,69]. As mentioned earlier, somatic cells preferentially use OxPhos for ATP production, whereas pluripotent cells rely on glycolysis as the main energy source, with less oxidative stress [51,70]. The successful reprogramming of somatic cells into iPSCs requires major changes in many aspects of cell function, such as gene expression pattern, epigenetic status, cellular structure, and, consequently, cell metabolism.

### 5.3. The Interplay between Core Transcription Factors at Different Stages of Reprograming

The major pluripotency-associated transcription factor OCT4 cooperates with other factors, such as SOX2 to drive somatic cell reprogramming. C-MYC acts as a catalyst in this process, increasing the efficiency of iPSC formation [23]. In particular, c-MYC regulates the expression of genes involved in glucose and glutamine catabolism in iPSCs [58]. It has been reported that the employment of high-glucose culture medium further increases the reprogramming efficiency [28], which occurs mainly through the enhancement of the glycolytic pathway. In particular, glycolysis upregulation affects the early steps of the reprogramming process [71]. Furthermore, *MYC* overexpression increases the mRNA and protein levels of glucose and glutamine transporters, as well as the expression of several enzymes involved in glycolysis, such as HK2, PFK, and LDH. c-MYC is critical to the occurrence of an initial hyperenergetic state of metabolism that is characterized by upregulation of both OxPhos and glycolysis [72,73,74]. This transient state of high energy production leads to an increased generation of ROS, which may act as secondary messengers. Consequently, the expression of numerous genes encoding transcription factors, such as nuclear factor (erythroid-derived 2)-like-2 (NRF2), nuclear factor kappa-light-chain-enhancer of activated B cells (NF-κB), and activator protein 1 (AP-1), is induced [75,76,77]. There are two possible functions of this transient state: (1) it has a major impact on gene expression status in cells undergoing reprogramming, which likely facilitates metabolic transition; (2) it may act as a response to the high metabolic energy demands during reprogramming. Subsequently, oxygen consumption via OxPhos is greatly reduced at a later stage of reprogramming, which is mediated by KLF4 [78,79]. Such mutual cooperation between transcription factors plays an essential role in successful reprogramming [79].

In reprogrammed cells, profound changes occur in the expression of genes encoding numerous metabolic enzymes. Upon metabolic reprogramming, the expression of glutaminase (GLS), an enzyme that catalyzes the first step in glutaminolysis, is upregulated [80]. This important adaptation allows highly proliferating cells, including PSCs, on the one hand, to acquire carbon flux via metabolic pathways to satisfy energy demands, and, on the other hand, to fine-tune the generation of intermediates, which are essential components for biosynthesis during proliferation [64]. The enhanced glutamine uptake also supplies the antioxidant glutathione (GSH) pool in PSCs to reduce oxidative stress.

### 5.4. Role of HIFs in the Metabolic Switch during Reprogramming

It has been shown that HIF1α and HIF2α are the key factors during iPSC generation, and their activity is required at specific stages of the reprogramming process [21,62,81]. Analysis of the signaling pathways triggered by the induction of pluripotency showed that activation of HIF1α and HIF2α occurs following a hyperenergetic metabolic state, because of the increased production of ROS and elevated expression of NRF2 [77]. As mentioned previously, HIF1α and HIF2α promote a metabolic switch in cells by increasing the expression of numerous metabolic, hypoxia-responsive genes [21,62]. Induction of HIF1α and HIF2α was sufficient to reduce oxidative metabolism in fibroblasts without the addition of reprogramming factors, which was mediated by changes in gene expression rather than changes in the number of mitochondria [21,62]. Interestingly, knockdown of HIF2α blocked the reprogramming process, and, similarly, extended HIF2α overexpression reduced reprogramming efficiency. This indicated that HIF2α is necessary for reprogramming, but only at a specific stage. The study by Mathieu, J. et al. demonstrated that the inhibitory effect of prolonged HIF2α expression on reprogramming efficiency is mediated by the TNF-related apoptosis-inducing ligand (TRAIL) by repressing caspase 3 activity [62]. Importantly, ESCs are not responsive to TRAIL, therefore this does not affect fully reprogrammed cells. Importantly, HIF1α and HIF2α are also present during reprogramming under normoxic conditions. However, the stability of HIF1α and HIF2α increases in hypoxia, boosting the reprogramming efficiency [35,62].

### 5.5. Remodeling of Mitochondria

Another important aspect of the metabolic switch during reprogramming to pluripotency is mitochondrial remodeling. The morphology of mitochondria changes from mature and abundantly present in somatic cells to immature, smaller, with low energy potential, and located in perinuclear zones in PSCs [82]. There are several mechanisms that may be involved in this transition. Firstly, mature mitochondria may be removed by mitophagy via an autophagy-related protein 5 (ATG5)-dependent and independent manner [83]. It has been shown that SOX2 factor is crucial to ATG5-dependent mitophagy through the inhibition of a mammalian target of the rapamycin (mTOR) pathway, which aids the progression of reprogramming [84]. It allows old mitochondria to be replaced by new ones with distinct characteristics. Secondly, the size of mitochondria can be reduced by active fragmentation. This process has also been observed in the early stage of reprogramming [74]. Moreover, the recent findings of Zhong et al. underline the importance of mitochondrial dynamics for the maintenance of pluripotency and the embryonic developmental potential of PSCs [85]. Mitochondrial function is determined by two dynamic processes: fission and fusion. An increase in fission activity results in mitochondrial fragmentation, whereas an augmented fusion activity leads to mitochondrial elongation. The proper balance between mitochondrial fusion and fission is of critical importance for the achievement of full pluripotency in PSCs. In particular, excessive mitochondrial fission may disturb the developmental potential of these cells [85]. The above-described mitochondrial changes lead to a complete switch in the metabolic state of the reprogrammed cells, resulting in the generation of iPSCs with peculiar metabolic properties. In this respect, mitochondrial respiration in pluripotent cells is limited to maintain their self-renewal. Accordingly, the expression levels of pluripotency markers were found to be downregulated in ESCs with an increasing copy number of mitochondrial DNA [67].

## 6. Metabolic Regulation of Pluripotency and Differentiation

Currently available data suggest that an important factor affecting PSC differentiation is oxygen abundance in the environment. Oxygen is consumed in cells mainly in mitochondrial OxPhos. Not only does it provide the cell with energy, but it also generates intermediates and ROS, which may finely regulate cellular metabolic transitions, thereby changing cell function and phenotype [67,86].

### 6.1. Mitochondrial Dynamics Regulates Pluripotency and Differentiation in PSCs

When PSCs start to differentiate, the number of mitochondria increases, and their morphology changes. In general, differentiated cells have more elongated mitochondria, with higher membrane potential, and, at the same time, the cells consume more O_2_ [87]. Importantly, increased mitochondrial activity leads to enhanced ROS generation. Thus, the number and activity of mitochondria are considered determining factors in the cellular response to oxidative stress [88]. Upon differentiation, dynamic changes in mitochondrial energy production occur, which affect differentiation propensity.

It has been shown that the generation and homeostasis of ROS constitute a regulatory mechanism of the transition from pluripotency to differentiation in PSCs. ROS, such as the superoxide anion (O_2_^−^), are abundantly formed by the partial reduction of oxygen in the mitochondrial electron transport chain (ETC) during aerobic respiration. The funneling of glucose carbon flux to mitochondria results in increased ROS formation, which above a particular threshold may act as secondary messengers in cells and trigger differentiation of iPSCs [89]. Activated glycolysis prevents such events by exerting protective effects in at least two ways. Firstly, by expanding the pool of NADPH generated in the PPP, and, secondly, by reducing the OxPhos pathway [59].

### 6.2. OxPhos and ROS Production Are Emerging Mediators of Stem Cell Shift from Quiescence to Differentiation

**Figure 4:**
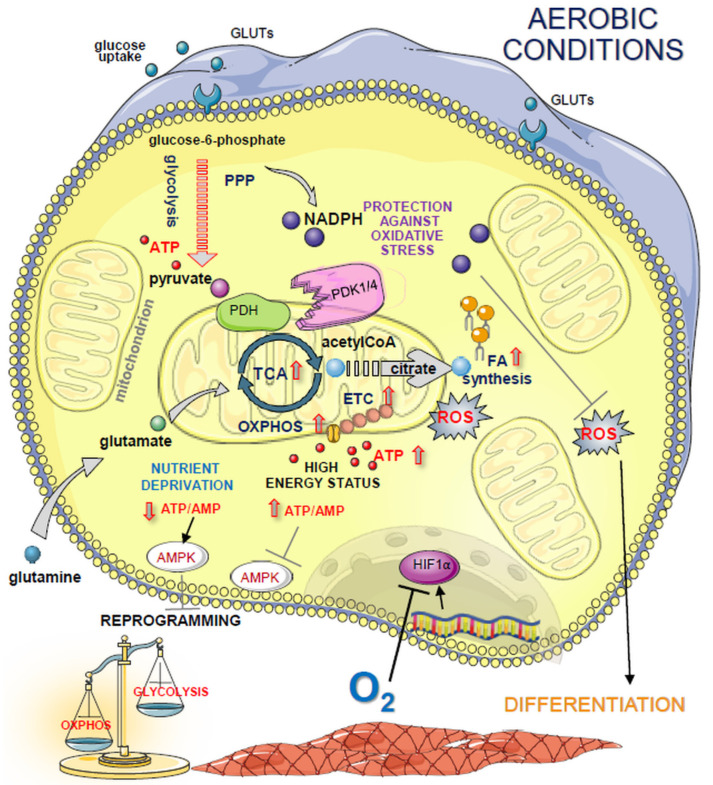
Outline of the cellular oxidative metabolism under normoxia. In O_2_ abundance, glucose catabolism to pyruvate is active. Next, pyruvate is transported to mitochondria to fuel the TCA cycle. The rate-limiting enzyme, PDH complex, is dephosphorylated and active. Glucose carbons form acetyl-CoA, and, as citrate, are transported to the cytoplasm for FA biosynthesis. ATP is produced mainly via OxPhos, with the generation of ROS. The continuous degradation of the HIF1α protein occurs under high oxygen levels. OxPhos favors spontaneous differentiation of PSCs. Abbreviations: AMP—adenosine monohosphate; AMPK—adenosine 5’-monophosphate-activated protein kinase; ATP—adenosine triphosphate; ETC—electron transport chain; FA—fatty acids; GLUTs—glucose transporters; HIF1α—hypoxia inducible factor 1α; NADPH—nicotinamide adenine dinucleotide phosphate; OXPHOS—oxidative phosphorylation; PDH—pyruvate dehydrogenase; PDK1/4—pyruvate dehydrogenase kinase 1/4; PPP—pentose phosphate pathway; ROS—reactive oxygen species; TCA—tricarboxylic acid.

When a cell starts to differentiate, the rate of glycolysis diminishes and a rapid switch to mitochondrial OxPhos occurs (Figure 4). The active OxPhos requires fueling the TCA cycle with glucose carbons. Mitochondrial pyruvate dehydrogenase complex (PDH complex, PDHC) catalyzes a rate-limiting step of feeding mitochondria with glucose carbons, namely, pyruvate decarboxylation. In the oxygen abundance, the activity of PDK1, which is an HIF1α downstream protein, decreases. The subsequent activation of PDHC results in the induction of mitochondrial aerobic metabolism, since the dephosphorylation and activation of PDH complex permits pyruvate to enter the TCA cycle instead of lactate production. In the mitochondrion, pyruvate supplies the TCA cycle for citrate synthesis, which, in the form of acetyl-CoA, is transported to the cytoplasm for fatty acid (FA) de novo synthesis [90]. As mentioned, the TCA cycle may also be supplied with glutamine carbons via α-ketoglutarate [58]. Mitochondrial TCA generates NADH, which, in turn, feeds the OxPhos and ETC to produce energy in the form of ATP. However, recent studies have indicated that in the cytoplasm/nucleus, the newly synthesized acetyl-CoA may have other distinct functions, depending on the metabolic context. It may play an important role in the induction of the differentiation program. The study of Cornacchia et al. demonstrated that the availability of exogenous lipids (an abundant source of acetyl-CoA) in culture media regulates the transition of pluripotency to differentiation of hiPSCs [91].

### 6.3. Mitochondrial Regulatory Proteins Influence Cell Differentiation and Maturation of iPSCs

Mitochondrial biogenesis is controlled by the expression of genes regulating oxidative metabolism, such as peroxisome proliferator-activated receptor γ co-activator 1α (*PGC-1α*). PGC-1α protein was reported to regulate mtDNA transcription and replication [92]. It is worth noting that PGC-1α may upregulate the expression of numerous genes encoding regulatory proteins of the mitochondrial metabolism by modulating the binding activity to specific sites in DNA of different transcription factors. PGC-1α governs the activities of cAMP response element binding protein (CREBP) and nuclear respiratory factors (NRFs). Such a change in the transcriptional program may play an important role at later stages of differentiation. Recently, Liu et al. investigated the effect of mitochondrial biogenesis on the metabolic maturation of hPSC-derived cardiomyocytes (hPSC-CMs) and found that PGC-1α promotes the mature phenotype of hPSC-CMs [93]. Since the immaturity of cultured hPSC-CMs during expansion still remains a significant and challenging problem, the modulation of cell function via PGC-1α may support acquiring the mature state of hiPSC-CMs for potential use in regenerative medicine.

### 6.4. Bioenergetic Status of iPSCs Regulates Pluripotency and Differentiation

Under oxygen abundance, when the respiration rate in mitochondria is high, a family of sirtuins (SIRTs), NAD-dependent deacetylases, acts as metabolic sensors of the NAD^+^/NADH level in the cell to precisely meet energy demands. Among SIRT proteins, SIRT1 may increase mitochondrial function and reduce oxidative stress. Induction of SIRT1 may also effectively delay age-related alterations linked to oxidative stress, which was demonstrated in murine and human ESCs upon enhanced ROS load [94]. Additionally, SIRT1 deacetylates the gene encoding the transcription factor SOX2 and regulates iPSCs reprogramming through the miR-34a–SIRT1–p53 axis. The precise role of particular SIRT proteins in the regulation of iPSCs reprogramming was extensively reviewed by Hsu et al. [95]. The study of Jing et al. showed that the oxidative phosphorylation activity and respiration capacity of undifferentiated iPSCs are low in contrast to iPSC-derived hepatocytes, which exhibit active mitochondrial metabolisms, including OxPhos and respiration. Jing et al. demonstrated that NAD can increase ATP levels and upregulate the expression of mitochondrial-encoded ETC genes, and that an elevation of intracellular NAD^+^ promotes SIRT1-mediated activation of peroxisome proliferator-activated receptor γ co-activator 1α (PGC-1α), a master regulator of mitochondrial biogenesis [96].

Stress conditions, such as glucose restriction, result in an energy deficit, which in turn activates the cellular master regulator of energy balance, adenosine 5’-monophosphate-activated protein kinase (AMPK). The report of Vazquez-Martin et al. demonstrated that pharmacological induction of AMPK activity using metformin reduces the reprogramming process of murine fibroblasts [97]. Interestingly, an increasing AMPK activity established a substantial barrier to reprogramming that could not be bypassed even by knockout of p53 protein. The activation of AMPK was accompanied by the downregulation of *Oct4* and changes in cellular bioenergetics [97].

### 6.5. Nitric Oxide Drives Differentiation of PSCs via Mitochondrial Function

Several studies have focused on the role of nitric oxide (NO) in the pluripotency/differentiation of PSCs. Tejedo et al. showed that NO at low concentrations may regulate stemness and may therefore be used for expansion of PSCs [98]. In compliance, NO maintains normal cellular ATP levels by inhibiting mitochondrial respiration (complex Ⅳ of ETC) and increasing glycolysis [92]. The molecular mechanism by which NO regulates PSC function remains unclear. However, the experimental data suggest that it controls cell fate mostly by mitochondrial function and endoplasmic reticulum stress. Interestingly, in vitro studies revealed a dual role of NO in the maintenance of pluripotency and differentiation, depending on its concentration. The exogenous exposure of mESCs to high concentrations of a NO donor promoted differentiation by downregulation of the pluripotency genes *NANOG* and *Oct4* [92].

### 6.6. Acetyl-CoA Plays a Role in the Differentiation of PSCs via Chromatin Epigenetic Regulation

Profound changes in the epigenetic state of chromatin occur during induction of pluripotency, as well as in the process of differentiation. Such epigenetic modifications include reversible chemical processes, such as DNA methylation/demethylation, and histone and chromatin remodeling. It was found that in ESCs, the promoters of pluripotency-associated genes are demethylated [23]. One of the epigenetic modifications is histone acetylation (by marking lysine residues), which is required to form euchromatin [90,99]. The induction of histone modification is an early event associated with the initiation of reprogramming, and it also plays a crucial role in the maintenance of pluripotency. The intracellular abundance of glycolysis-derived acetyl-CoA is critical for controlling histone acetylation in the nucleus [100]. It was shown that the glycolytic switch can prevent differentiation and maintains the pluripotency of PSCs by providing high cytoplasmic concentration of acetyl-CoA. A recent report by Moussaieff et al. aimed to elucidate how a glycolytic-OxPhos switch triggers the loss of pluripotency with respect to the earliest steps of differentiation [101]. The authors investigated the initial metabolic changes induced during the first hours of spontaneous differentiation of PSCs. They demonstrated a rapid decrease of glycolytic flux to form cytosolic acetyl-CoA in the early steps of differentiation. It resulted in a loss of histone acetylation and a consequent loss of pluripotency. These data highlight the important role that glycolysis-derived acetyl-CoA plays in maintaining the balance between pluripotency and differentiation in PSCs. The study of Carey et al. also reported that epigenetic and genetic modifications may be extensively regulated by particular metabolites via metabolic reprogramming [102]. In naïve mouse ESCs, histone DNA demethylation and resulting pluripotency maintenance were promoted by an elevated α-ketoglutarate-to-succinate ratio. Such epigenetic marks, including histone acetylation and methylation, are regulated by metabolite abundance and control PSCs fate.

## 7. Oxygen Availability and Differentiation Pathways in PSCs

### 7.1. Oxygen Acts as a Morphogen during Development

**Figure 5:**
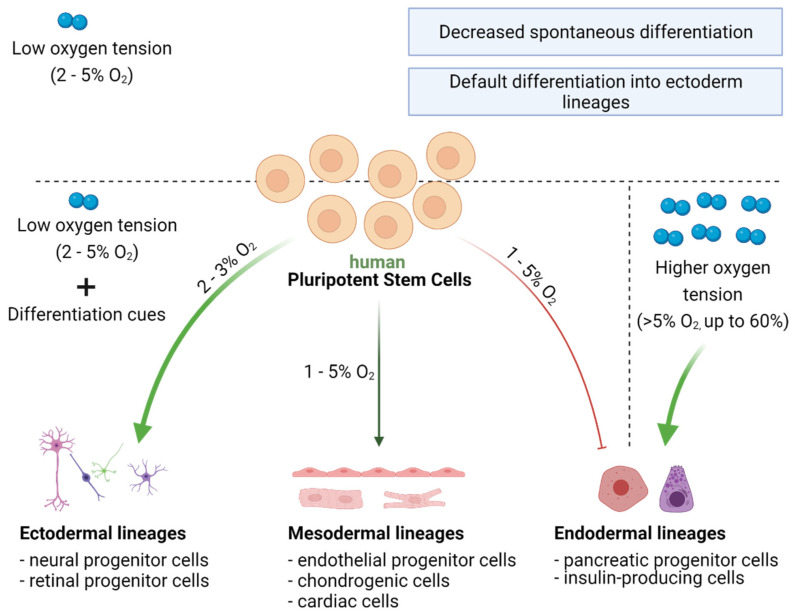
Impact of oxygen on the differentiation of hPSCs. hPSCs can differentiate into cells of three germ layers: ectoderm, mesoderm, and endoderm. Oxygen concentration influences the differentiation capacity of hPSCs. Low oxygen tension alone decreases spontaneous differentiation of hPSCs; however, in combination with differentiation cues, hypoxia facilitates differentiation into endodermal and mesodermal lineages. Conversely, differentiation of hPSCs into endodermal lineages decreases with low oxygen tension and increases under high oxygen concentrations (up to 60%).

During embryonic development, oxygen likely acts on cells as a morphogen. Low oxygen concentration (3–5%) in the early stage of embryogenesis supports the pluripotent state of ESCs. At a later time, because of the formation of the blood vessels and the circulatory system, oxygen concentration increases. As a consequence, an oxygen gradient is generated in the developing embryo, which, in combination with other stimuli, influences cell fate and differentiation pathways. The importance of oxygen for proper embryo development has been demonstrated in animal studies. Mice lacking HIF1α were characterized by embryonic lethality at embryonic day E10.5, accompanied by defects in vascularization and neural tube formation [103,104]. Furthermore, mimicking in vivo differentiation conditions in the in vitro cell culture of hPSCs confirms that oxygen is an important factor to consider when optimizing differentiation protocols [105,106]. Interestingly, hypoxia was shown to have a dual effect on hPSC differentiation. On the one hand, hypoxic conditions prevent the spontaneous differentiation of hPSCs [19,36,40,46] and support the expression of pluripotency markers [19,20,36,40]. On the other hand, however, low oxygen tension combined with the activity of defined factors present in the cellular mileu may facilitate differentiation into specific cell lineages [106,107,108,109,110,111,112,113,114,115] (Figure 5).

### 7.2. Directed Differentiation of hPSCs Can Be Modulated by Oxygen Concentration

PSCs can differentiate into cells of three germ layers: ectodermal, mesodermal, and endodermal. Briefly, cells of the ectodermal lineage give rise mainly to neural cells and the skin epithelium. Cells of the mesodermal lineage can differentiate into muscle, kidney, bone, cartilage, red blood cells, endothelium of blood vessels, or adrenal cortex. Cells of the endodermal lineage form the respiratory tract, bladder, liver, and pancreas. As studies show, the lineage commitment of hPSCs is strongly influenced by oxygen concentration. Hypoxia in particular seems to favor ectodermal differentiation, but also facilitates the differentiation into cell types of mesodermal origin, e.g., endothelial cells and cardiomyocytes (CMs). In contrast, low oxygen was shown to restrict endodermal lineage specification [105,106,107,108]. These observations agreed well with data from embryonic development. Accordingly, one of the first events during embryogenesis is the formation of blood vessels to properly nourish the embryo, these are composed of endothelial cells from the mesodermal lineage [109]. The development of the neural system also occurs early during embryogenesis. Knockdown of HIF1α significantly impairs neurogenesis, indicating that hypoxia is an important factor in ectoderm differentiation [103,104]. In contrast, knockdown of HIF1α promotes endoderm differentiation, confirming that endodermal lineage specification is facilitated under higher oxygen concentrations [108]. Because of differences in physiological properties and molecular backgrounds among PSCs from different species, the following section will only focus on cells of human origin.

#### 7.2.1. Ectoderm Differentiation

The current state of knowledge suggests that hypoxia not only promotes ectoderm differentiation but also points to the ectoderm as the default differentiation pathway, as demonstrated in both hESCs and hiPSCs [106,107]. Efficient differentiation of hPSCs into neural and retinal cells is particularly desirable, considering their potential use in cell replacement therapies. Importantly, such cells may also serve as a model system to study the development of neurodegenerative diseases, such as Alzheimer’s or Parkinson’s disease. Several studies have investigated further these differentiation pathways. Stacpoole et al. found that differentiation of hESCs under hypoxia (3% O_2_) increased the number of neural progenitor cells (NPCs) [108]. Moreover, transcriptome profiling performed on hESC- and hiPSC-derived embryonic bodies (EBs) revealed that culture under 2% oxygen induced the expression of genes involved neural differentiation, which is mediated by HIFs. Interestingly, hypoxia also affected later stages of neural differentiation by changing the bias of NPCs toward becoming glial cells instead of neurons [106]. The above studies showed that low oxygen concentration (2–3%) promotes the differentiation of hPSCs into NPCs. Importantly, this effect was not observed in a recent study examining neural differentiation of hiPSC-derived EBs under 5% oxygen [111]. This could result from a decreased efficiency in EB formation under hypoxia, which was observed during the experiment. The difference may also be explained by the higher oxygen concentration (5% O_2_) applied in this study. In addition, cell line specific differences should also be considered, as only one hiPSC line was examined [111].

The effect of oxygen availability on the efficiency of retinal cell differentiation has also been examined. It was shown that 2% O_2_ increased the expression of genes defining early eye field, including SIX homeobox 3 (*SIX3*) and LIM homeobox 2 (*LHX2*), in the EBs derived from both hESCs and hiPSCs, in comparison to 20% O_2_ [112]. This effect was followed by an increase in the number of retinal progenitor cells (RPCs) in the later phase of differentiation. The obtained cells were characterized by the expression of transcription factors: paired box 6 (PAX6) and Ceh-10 homeodomain-containing homolog (CHX10), which are necessary for their proliferation and the maintenance of multipotency. Interestingly, the efficiency of the differentiation protocol toward RPCs was cell line-dependent—lower efficiency was achieved in case of hESCs compared to hiPSCs. Nonetheless, increased RPC generation rates in low oxygen conditions were noted for both these cell types [112].

#### 7.2.2. Mesoderm Differentiation

A growing body of evidence suggests that low oxygen concentration, in combination with appropriate differentiation stimuli, promotes hiPSC differentiation into cells of mesodermal origin, such as endothelial cells, CMs, or chondrocytes [105,113,114,115]. The effect of low oxygen tension on the endothelial differentiation potential of hPSCs was extensively reviewed in a recent paper by Podkalicka et al. [116]. Briefly, the available data indicated that endothelial specification is enhanced under hypoxia. For example, it was shown that 1 and 5% O_2_ (compared with 21%) enhanced the differentiation of hESCs into endothelial progenitor cells, as evidenced by a significant upregulation of transcript levels for the vascular endothelial growth factor isoform A (*VEGF-A*) and B (*VEGF-B*), after 5 days of differentiation. This was accompanied by an increase in the number of cells expressing the VEGF protein and angiopoietin-like 4 (ANGPTL4), which are crucial factors regulating endothelial cell function [105].

The beneficial effect of hypoxia was also observed in cardiac differentiation of hPSCs. Low oxygen concentration (4%) significantly increased the number of hESC-derived contracting EBs. This was also supported by upregulated expression of early and late cardiac-specific genes [113]. Our own data confirmed these observations. We showed that hiPSC pre-conditioning at physiological oxygen level (5% O_2_) enhances generation of CMs with the elevated transcript levels of genes expressed by mature CMs, in comparison to cells generated solely in normoxia [117]. Such results were further supported by Burridge et al., who demonstrated that 5% oxygen promoted cardiac differentiation of both hESCs and hiPSCs [115]. The authors obtained a higher number of contracting EBs in reduced oxygen condition, likely mediated by the WNT signaling pathway. Importantly, the observed effect was cell type-specific, as cardiac differentiation efficiencies for hESCs were already high (>90%), and the advantageous effect of hypoxia was mainly observed in hiPSC lines [115].

Within mesodermal lineages, the effect of oxygen was also examined on the chondrogenic differentiation capacity of hESCs. Cartilages, composed of chondrocytes, are avascular tissues located in hypoxic niches. Therefore, mimicking the natural environment by reducing oxygen concentration to 2% enhanced chondrogenesis in hESCs. This resulted in increased collagen II production and improved biomechanical properties, including a higher tensile strength modulus and increased compressive properties of the tissue engineered in hypoxia [114].

#### 7.2.3. Endoderm Differentiation

The effect of hypoxia on the endodermal specification of hPSCs, in contrast to other lineages, appears to be unfavorable [107]. However, studies addressing this issue are limited, and most of them investigated hiPSCs. Hypoxia (2% O_2_) was shown to cause a decrease in the expression of endodermal markers in EBs [107]. In addition, knockdown of HIF1α enhanced endoderm differentiation [107]. In agreement with the above results, high oxygen concentration (60%) was shown to significantly enhance the differentiation of hiPSCs into pancreatic progenitors and insulin-producing cells, both of endodermal origin. Cell culture under 60% O_2_ from d3 to d7 of the differentiation process increased neurogenin-3 expression, which is a marker of the endocrine progenitors that give rise to the pancreas. Moreover, the effect of high oxygen pressure resulted in extended to enhanced insulin production in the differentiated cells, and was mediated by reduced HIF1α signaling [118].

## 8. Oxygen in Cancer Stem Cells

### 8.1. PSCs and CSCs—Are They Distinct or Similar?

**Figure 6:**
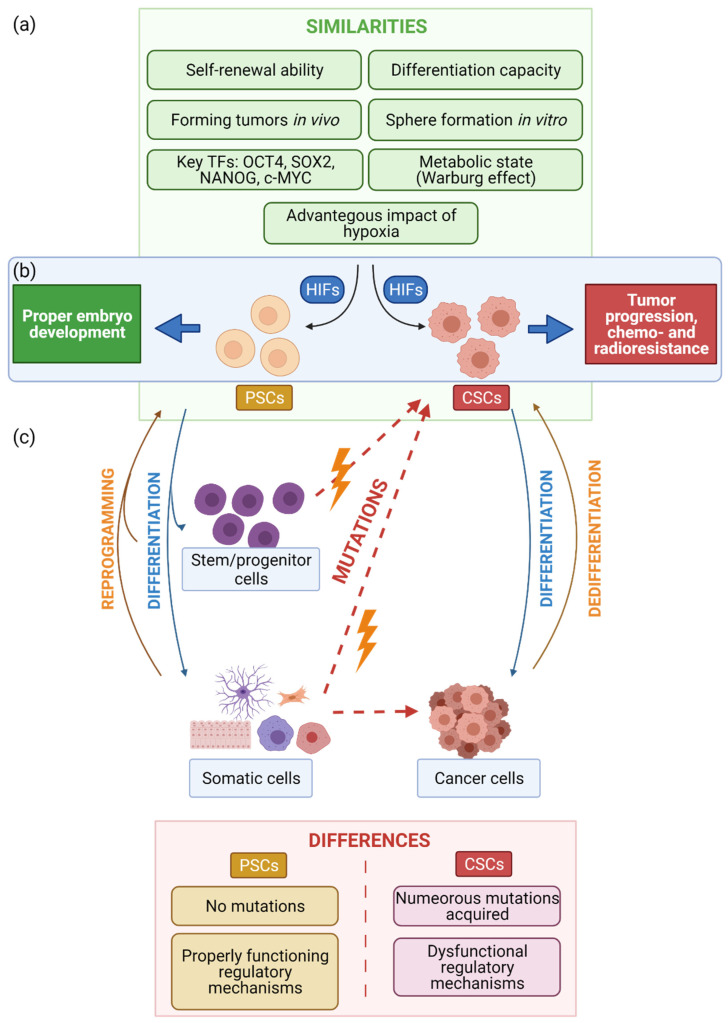
Comparison of PSCs and CSCs. (**a**) Similarities. Both PSCs and CSCs can self-renew and differentiate into other cell types. They can both form spheres in vitro and tumors in vivo. Their function is regulated by a similar set of transcription factors (OCT4, SOX2, NANOG, c-MYC), and they both rely on glycolysis as the main source of ATP production. (**b**) Diverse impact of hypoxia on PSCs and CSCs. Although oxygen concentration is an important factor for the function of both CSCs and PSCs, it leads to distinct effects. In the case of PSCs, hypoxia supports the state of pluripotency and proper embryo development. In contrast, a low oxygen condition in CSCs promotes tumor progression and radio- and chemoresistance. (**c**) Substantial differences between CSCs and PSCs. The regulatory mechanisms in PSCs are functional. In contrast, such mechanisms are dysregulated in CSCs due to mutations, which drive further mutagenesis and excessive proliferation.

CSCs are a subpopulation of cancer cells, able to drive cancer growth and regenerate tumors. Thus, CSCs are mostly responsible for cancer resistance to therapies. They have been identified in many types of cancers, including: hematopoietic, colon, lung, breast, liver, brain, ovarian, pancreas, prostate, melanoma, bladder, head and neck [119,120]. Although the CSCs found in different tumors may differ, they are all defined as cancer-initiating cells that possess a high capacity to self-renew and differentiate into cancer cells. Moreover, they can generate tumors when implanted into immunodeficient mice [121]. Such a description strongly resembles PSCs, which can also self-renew, differentiate into other cell types, and form teratomas in vivo [4]. Interestingly, CSCs share many other characteristics with PSCs (Figure 6). Both CSCs and PSCs can form spheres (called EBs for PSCs) in in vitro culture, which is recognized as an indicator of their proliferative and differentiation capacities [4,122]. Moreover, the similar set of transcription factors seems to be crucial to their function. In both CSCs and PSCs, transcription factors such as OCT4, SOX2, NANOG, or c-MYC are highly expressed and necessary for the maintenance of the stem cell-like phenotype [4,123,124,125]. Notably, they exhibit similar metabolic profiles and rely on glycolysis as a main source of ATP production, even in the presence of oxygen [51,126,127]. However, despite the abovementioned similarities, it is also important to underline the intrinsic difference between PSCs and CSCs, which is crucial to their function. While in PSCs, vital processes such as proliferation and differentiation are strictly controlled by the appropriate regulatory mechanisms, these processes are impaired in CSCs because of mutations or chromosomal rearrangements. Consequently, the high mutation rate leads to uncontrolled proliferation and differentiation of CSCs, triggering tumor growth.

Moreover, CSCs are characterized by continued mutagenesis, which creates new phenotypes that are difficult to control. Most of the cells accumulate novel mutations and are able to escape apoptosis, which makes them an extremely difficult target for anti-cancer therapies. On the other hand, PSCs display high basal levels of proteins involved in the DNA damage response (DDR), which secures the stability of their genome [128,129]. In particular, these cells more frequently utilize the faithful homologous recombination (HR) pathway to repair double strand DNA breaks (DSBs) than do somatic cells [130,131]. Therefore, excessive DNA damage or abnormal stress more likely triggers apoptosis in PSCs via the p53-dependent pathway. Importantly, p53 protein is upregulated in PSCs, which constitutes a “safety switch” and keeps the threshold of mutation accumulation low. Thus, dysfunctional or damaged PSCs are rapidly eliminated from a population, preventing the spread of mutations that may disturb proper development. Interestingly, as was already described for PSCs, hypoxia also seems to be an essential factor influencing the behavior of CSCs [132,133].

### 8.2. Hypoxia Triggers Aggressive Phenotypes of CSCs

The important role of oxygen concentration in regulating PSC and CSC functions is another common feature of these cells. They both reside in hypoxic niches and the beneficial effect of hypoxia was observed for both through the induction of HIFs [134]. However, this influence leads to distinct consequences. In the case of PSCs, activation of HIFs in low oxygen concentration fosters the state of pluripotency but also impacts differentiation pathways. Both these states are necessary for proper embryo development. Conversely, the advantageous effect of hypoxia on CSCs results in tumor growth and the acquisition of new phenotypes, which may lead to chemo- or radioresistance. For instance, it was observed that hypoxia inhibits the activation of p53 and p16 proteins in CSCs through the activity of HIFs, restraining tumor cell death [135]. Interestingly, the research points to HIF2α as the major mediator of the supportive effects of hypoxia on CSCs, similar to PSCs. In CSCs, activated HIF2α inhibits p53 and simultaneously increases the transcription of *c-MYC*, thus accelerating CSC proliferation [136,137]. Moreover, acute hypoxia (in a range of 0.1% O_2_) and anoxia (lack of oxygen) are associated with the increased aggressiveness of cancer cells [138,139]. It has been shown that acute hypoxia decreases expression of proteins involved in the HR pathway in cancer cells, thereby promoting genome instability [138]. In addition, HIF activity in a hypoxic environment triggers the expression of a variety of genes crucial to tumor progression and migration [140]. Therefore, the HIF pathway strongly influences the behavior of CSCs, which makes it an excellent target for an anti-cancer therapy [141,142,143].

## 9. Conclusions

Human PSCs constitute a very promising cell source for both science and medicine. They can serve as a platform for personalized drug testing or cell replacement therapy. It is therefore important to ensure their genome stability, undisturbed pluripotency profile, and robust multidirectional differentiation potential, particularly during prolonged cell culture. A crucial factor in the PSC microenvironment that substantially impacts their characteristics is oxygen abundance. While the most commonly applied O_2_ level for PSC culture equals the concentration in the air, it remarkably exceeds the physiological oxygen level present in tissues and during embryo development. Accordingly, mild hypoxia (5% O_2_) has been shown to exert an advantageous effect on most of the processes regulating PSC functions, such as metabolism, proliferation, the maintenance of pluripotency, and differentiation. Importantly, oxygen concentration can positively regulate the process of somatic cell reprogramming. Additionally, low oxygen concentration supports the physiological metabolic state of hPSCs, which relies on glycolysis as the main source of ATP production. In such conditions, glycolytic reactions occur with minor production of ROS, controlling cell differentiation, reducing the risk of oxidative DNA damage, and keeping a high cytosolic acetyl-CoA level for the maintenance of cell pluripotency. However, further studies are needed to fully understand the molecular machinery that governs PSC activity, particularly in cell culture conditions resembling the physiological environment. Such knowledge can further enhance the utility of PSCs in biomedical applications, and, importantly, increase the safety of future iPSC-based therapies.

## Figures and Tables

**Table 1:** The impact of hypoxia on pluripotent stem cells. The table describes changes observed under hypoxia compared to normoxia conditions. Abbreviations: ↑—upregulated; ↓—downregulated; ↔—unchanged; d—days; H—hypoxia; hESCs—human embryonic stem cells; hfF—human foreskin fibroblasts; hiPSCs—human induced pluripotent stem cells; MEFs—mouse embryonic fibroblats; Ma—Matrigel; m—month; nd—no data; p—passage.

Cell Type	No. of Cell Lines	% O_2_	Duration of H	Substrate	Sponta-Neous Differen-Tiation	Cell Growth	Markers of Pluripo-Tency	Additional Remarks	Ref.
hESCs	1	1; 3; 5	15 d	MEF; Ma	↓	↓	nd	enhanced EBs formation	[36]
hESCs	1	5	3 p	MEF; Ma	↓	↑	↑	HIF2α as the most important regulator in H	[40]
hESCs	3	4	7 d	hfF	↓	↑	↑/↔	increased MYC expression via HIF2α	[19]
hESCs	3	2	10 p	Ma	nd	nd	↔	302 genes up in H (transcriptome analysis)	[50]
hESCs	3	2	5 p	Ma	nd	↑	↔	smaller and less granular cells; reduced chromosomal aberrations	[37]
hESCs	2	5	7; 14; 28 d	MEF	↓	↓	↔	-	[46]
hESCs	2	4	10 p	MEF; Ma	↔	nd	↔	-	[49]
hiPSCs	2	2.5; 5	2 m	MEF	nd	nd	↑ NANOG ↓ OCT4	53PB1 down	[48]
hiPSCs	1	5	14 d	MEF	↓	↑	↑	HIF2α activated in H	[20]
